# The impact of smart sports services on residents’ sports participation: the mediating role of exercise self-efficacy and perceived usefulness

**DOI:** 10.3389/fpubh.2026.1786175

**Published:** 2026-04-01

**Authors:** Yu Cong Huang, Liang Ming Li

**Affiliations:** School of Business, Hunan University of Science and Technology, Xiangtan, China

**Keywords:** multiple intermediary mechanisms, perceived value, personalized service, smart sports, sports participation

## Abstract

**Objective:**

This study aims to explore how smart sports services influence residents’ sustained sports participation, with a focus on the mediating roles of perceived usefulness and exercise self-efficacy.

**Methods:**

Drawing on social cognitive theory and the technology acceptance model, this study constructs an extended theoretical framework incorporating service personalization and perceived value. A questionnaire survey was administered to 354 urban residents with prior experience in smart sports services across four first-tier cities in China. Data were analyzed using structural equation modeling via SPSS 27.0 and Amos 28.0.

**Results:**

(1) Service personalization and perceived value are significantly positively associated with sports participation; (2) Perceived usefulness and exercise self-efficacy partially mediate the relationship between service personalization, perceived value, and sports participation; (3) A significant chain mediation effect is observed, wherein service personalization enhances perceived usefulness, which in turn strengthens exercise self-efficacy, ultimately promoting sustained sports participation.

**Conclusion:**

The findings reveal a sequential psychological mechanism—“technology perception → cognitive evaluation → efficacy belief → behavioral outcome”—through which smart sports services facilitate residents’ sports engagement. These insights provide theoretical support for optimizing smart sports service design and inform policy aimed at promoting digital health interventions.

## 1 Introduction

Premier Li Qiang explicitly stated at the State Council executive meeting in 2024 that “digital technology should be used to empower the health of all citizens, promote the deep integration of smart sports services into the daily lives of the people, and truly transform technological dividends into a sense of health gain” (1) Central People’s Government of the People’s Republic of China. State Council executive meeting makes significant deployment on digital economy, accelerating the digitalization of industries and the industrialization of digital technology. This strategic deployment is highly consistent with the goal of “deep integration of mass fitness and health” in the “Healthy China 2030” Planning Outline, and it is also a deepening implementation of the requirement in the “National Fitness Program (2021–2025)” to “improve the accessibility of sports services through digital means”.

In 2022, the total scale of China’s sports industry reached 336 million yuan. The market size of smart sports has reached 98.1 billion yuan. Against this backdrop, as an emerging trend in the sports industry, smart sports not only have achieved remarkable results in promoting personal health and enhancing competitive levels but also have driven the upgrading of the sports industry and the wide dissemination of sports culture, demonstrating strong growth momentum and broad market prospect (2).

However, the current level of residents’ sports participation in China still faces severe challenges: although the proportion of people who frequently participate in sports exercises increased to 37.2% in 2023, only 26.5% of them have used smart sports services, and the continuous usage rate of users is less than 40%. This contradiction reveals two major practical problems: first, there is a structural mismatch between the technological supply of smart sports services and the actual needs of residents, such as the redundancy of functions in smart fitness equipment while neglecting the emotional resonance of user experience; second, residents’ perception of the value of smart sports services has not been effectively transformed into continuous participation behavior, and problems such as low trust in technology and concerns about data privacy are widespread (3). Taking a smart fitness venue in a first-tier city as an example, the usage rate of its technical modules such as physical fitness testing equipment and VR sports courses is only 52%, and user feedback shows that “service homogenization” and “unclear value perception” are the core reasons for giving up usage (2).

This phenomenon confirms the academic criticism of the “technology piling trap”: if smart sports only focus on hardware upgrades while ignoring the psychological mechanisms of users, it will be difficult to break the dilemma of “high investment and low stickiness.” Therefore, exploring how smart sports services drive residents’ continuous participation is not only a practical need for implementing the national “digital and real integration” policy but also an urgent need to fill the theoretical gap between technology acceptance and behavioral transformation.

## 2 Literature review

Compared with traditional sports services, the core difference of smart sports services lies in the fundamental transformation of service paradigms and operational logics. The traditional model mainly relies on physical sports facilities and manual management, with service content presenting static and homogeneous characteristics. Residents have to passively adapt to the fixed opening hours of venues and standardized course arrangements, and there is a significant temporal and spatial mismatch between service supply and demand. Meanwhile, the assessment of exercise effects mostly depends on subjective feelings, lacking objective data support, making it difficult to form positive incentives for continuous participation. Smart sports services, as a practical carrier of digital technology empowering mass fitness, are essentially user demand-oriented, integrating sports resources through big data, Internet of Things, and artificial intelligence technologies to provide personalized, precise, and sustainable sports participation support systems (4). With the deep integration of information technology in the sports field, smart sports services are gradually reshaping residents’ sports participation patterns.

### 2.1 Service personalization

Service personalization, was first introduced by the existentialist philosopher Sartre in the 1940s, referring to the comprehensive relationship formed by an individual’s multiple intentions toward an object (5). The ownership relationship between customers and service offerings is the foundation for understanding customer psychology and behavior. Service Personalization, as a concept based on marketing theory and practice, focuses on the phenomenon of ownership relationships in the relationship between customers and the services provided to them. Personalization is the high-quality, deep connection established between users and the services they own, representing the psychological process and internal state of users internalizing services as their own. Brown and Cunningham, using purchase quantity, frequency, and market share as observation points, found that consumers exhibit continuous purchasing behavior based on product characteristics and personal preferences during the purchasing process (6, 7), which is consistent with the ultimate goal of smart sports services, that is, to achieve long-term stability and continuous investment through physical exercise to obtain physical and mental satisfaction.

### 2.2 Perceived value

The social information processing theory holds that an individual’s activities and behaviors do not occur in a vacuum but are usually influenced by complex and ambiguous social contexts (8). The social environment in which an individual is situated provides various information that affects their attitudes and behaviors. During the process of receiving external information, individuals form certain cognitive evaluations through self-representation and other means, and use these as cues to adjust their subsequent attitudes and behaviors (9).

Perceived value can be roughly divided into two dimensions (10). The first is functional perceived value, which means that the products or services provided by enterprises must meet the needs and expectations of customers in terms of quality, performance, and performance. The second is emotional value, which refers to consumers’ preferences and evaluations of the degree to which the perceived activity process meets their emotional needs. Compared with the functional aspects of the activity process, consumers are more concerned with higher-level needs such as spiritual pleasure, self-actualization, and emotional satisfaction. Based on the “equity theory,” American scholar Valarie A. Zeithaml (5) pointed out that perceived value is a concept ultimately measured by consumer interests. Compared with objective activity performance, perceived value can reflect consumers’ more real and vivid feelings.

### 2.3 Exercise self-efficacy

Self-efficacy (11), proposed by Bandura in his social cognitive theory, describes an individual’s confidence in their ability to successfully complete a task or achieve a goal in a specific situation. According to Bandura, self-efficacy is the central mechanism through which individuals exercise control over their motivation, thought processes, and behavior—particularly when facing obstacles or challenges. Sallis and Hovell’s research indicates that self-efficacy is the most closely related variable to exercise behavior. In the context of smart sports services, exercise self-efficacy serves as the critical bridge between external technological stimuli and internal behavioral motivation. When users perceive that smart sports technology can help them achieve exercise goals, their confidence in their own ability to persist in exercise is enhanced, which in turn drives sustained participation. This positioning of exercise self-efficacy as a core mediator is theoretically grounded in social cognitive theory’s triadic reciprocal determinism model, where environmental factors, personal cognitive factors, and behavior mutually influence each other.

Second, exercise self-efficacy possesses unique functional characteristics that make it particularly suitable as a mediator: it is task-specific (referring to confidence in performing specific exercise tasks), context-dependent (adjusting to changes in exercise environment), and dynamic (evolving with exercise experience). Most importantly, self-efficacy is malleable and can be enhanced through four sources identified by Bandura: mastery experiences, vicarious experiences, verbal persuasion, and physiological/affective states. Smart sports services, through their personalized features, can activate all four sources, making self-efficacy a theoretically sound and practically relevant mediating variable.

Third, the specific context of physical activity demands a focus on self-efficacy. Unlike passive technology acceptance, sports participation requires sustained effort, overcoming physical discomfort, and persisting through obstacles—exactly the conditions where self-efficacy plays its most crucial role.

### 2.4 Perceived usefulness

From the perspective of social cognitive theory, perceived usefulness is essentially a value judgment. It constitutes the core cognitive basis of users’ perceived value. This theory emphasizes that after evaluating environmental cues, individuals form expectations of the potential outcomes of behavior. Perceived usefulness is precisely the user’s expectation of the efficacy of “using this technology will bring positive results.” Based on this expectation and value judgment, individuals will prioritize those behaviors that they perceive as generating greater utility for themselves. Therefore, the greater the perceived usefulness, the greater the likelihood that an individual will take the corresponding action. This process reflects the driving role of cognitive factors on behavioral motivation and choice in social cognitive theory. The research by Xu Guanglu further supports this point, indicating that perceived usefulness can stimulate an individual’s intrinsic motivation. When an individual’s perceived usefulness of a specific thing or behavior is higher, the degree to which their intrinsic motivation is activated is stronger, and they are more inclined to take that action or take positive steps to achieve their goals (12).

### 2.5 Sports participation

Sports participation refers to the behavior and process in which individuals or groups actively or passively engage in sports activities, sports organizations, sports events, and related activities. This concept not only includes individual participation in physical activities such as exercise and competitive games but also encompasses social sports activities, cultural consumption, and sports social interaction, among other multi-dimensional aspects. The core of sports participation lies in individuals achieving physical and mental health, social integration, and self-value enhancement through sports activities, and it also reflects the influence of social structure, economic conditions, educational levels, and other factors on individual sports behavior. From a sociological perspective, sports participation is not only an individual behavioral choice but also a manifestation of social stratification, cultural capital, and health equity.

### 2.6 Literature summary

Through the research and review of domestic and foreign literature, there is still room for improvement in current studies on the impact of smart sports investment on residents’ participation in physical exercise: (1) Most of the current research focuses on the usage of smart devices, while there is a lack of exploration into the psychological and state changes of users themselves, and there is a lack of research on the influence of subjective factors after residents’ exercise. (2) Existing literature has confirmed in multiple dimensions that there is a connection between physical exercise behavior, exercise self-efficacy, behavioral cognition, and behavioral attitude, but no study has delved into the influence and connection among different variables within psychological factors. Due to the existence of numerous external interference factors between sports services and actual usage experiences, a unified evaluation standard has not yet been formed in the academic circle. (3) In empirical research, due to the wide range of content involved, it is difficult to refine research indicators within a certain range, and the interaction relationship among various factors at different exercise levels has not been verified and analyzed, making it impossible to determine the influence path of each factor. Therefore, this study divides smart sports services into two specific dimensions conducive to quantitative research: service personalization and perceived value. Among them, service personalization is regarded as the actual feeling factor of smart sports services, considering the substantive impact of sports services; perceived value is regarded as the objective factor of smart sports services, considering the personalized evaluation of sports services due to individual subjective factors. At the same time, exercise self-efficacy and perceived usefulness are selected as internal factors influencing residents’ sports participation. Verify the theoretical hypotheses through questionnaires and data analysis results, provide targeted suggestions for the practical implementation of smart sports services.

## 3 Research hypotheses

### 3.1 Service personalization and sports participation

In the context of smart sports services, Service personalization essentially involves achieving dynamic adaptation between service supply and user demand through technological empowerment, which is manifested as precise output under the trilateral interaction of “technology-user-scene.” On the one hand, it collects users’ physiological data in real time through Internet of Things technology and forms personalized exercise plans through AI algorithms; on the other hand, digital twin technology builds user digital profiles and intelligently schedules service resources based on spatio-temporal scene characteristics. This service model breaks through the service limit provided by the traditional “standardized supply” sports services and deeply embeds digital technology into users’ life scenarios, thereby creating a “thousand faces for a thousand people” service ecosystem. Therefore, whether the connection between service personalization and user behavioral intention is close has become an important indicator for measuring the effectiveness of sports services. The application of smart sports technology can break through the “one-to-one” boundary of traditional sports service methods, allowing services to penetrate deep into customers’ hearts and generate a sense of ownership beyond property rights over products/services (5). In summary, Service personalization, as a technical indicator, can not only be quantified but also provide a reference at the psychological level for “whether users are satisfied with the service.” The pathway from service personalization to sports participation operates through a sequential psychological mechanism. First, personalized services enhance users’ perception of the technology’s usefulness by demonstrating its ability to meet individual needs. This increased perceived usefulness subsequently strengthens users’ exercise self-efficacy—their confidence in overcoming obstacles and persisting in physical activity. Ultimately, this reinforced efficacy belief translates into sustained sports participation behavior. This “technology characteristic → cognitive assessment → efficacy belief → behavioral outcome” pathway aligns with the core tenets of social cognitive theory, which posits that environmental factors influence behavior through cognitive mediators. Based on this, this paper proposes the research hypothesis:

*H1*: Service personalization is positively correlated with sports participation.

### 3.2 Perceived value and sports participation

Creating perceived value for consumers has a direct and indirect positive impact on consumers’ brand loyalty (13). In the field of sports services, consumers’ loyalty to a sports service technology directly affects their sports behavior, manifested as: the maturity of sports service technology is conducive to forming a stable sense of value gain, and the higher the perceived value level formed, the more frequently they participate in sports exercises (14). In real situations, individuals’ motivations for participating in sports activities are often dynamic and easily disturbed. Smart sports technology, through its high perceived value, not only enhances users’ immediate emotional value during the usage process but also strengthens their sense of gain (15) by facilitating visible actual results These multi-dimensional positive feedbacks work together to provide a strong realistic support for individuals to form stable and lasting participation motivation.

In addition, Lei Xiaoying (16) found that when the demand for a technical service matches an individual’s emotional value, participation willingness can have a significant positive impact on participation behavior. This means that good perceived value can, to a certain extent, make up for the lack of objective material conditions, allowing individuals to still have a good sports experience even with lower material support. Therefore, in the context of smart sports services, perceived value is reflected in the fact that emotions or feelings are aroused when participating in sports interactions, thereby bringing mental pleasure, satisfaction and realization of personality, and satisfaction of emotional value.

Based on this, this paper proposes the research hypothesis:

*H2*: Perceived value is positively correlated with sports participation.

### 3.3 The mediating role of perceived usefulness

Perceived usefulness is manifested in the interaction between service personalization and perceived value as follows: through the integrated sensor control system, audiences can interact with the competition, significantly enhancing the immersion, interest and sense of control in watching the game (17). This not only strengthens users’ perception of the usefulness of the technology, but also significantly enhances the overall perceived value of smart sports services by providing unique, pleasant and controllable experiences. In this process, it first meets users’ expectations for functional utility, and then shapes a high-level multi-dimensional perceived value. These dual cognitions work together to strengthen users’ specific perception and positive attitude toward sports events, creating favorable cognitive conditions and internal driving forces for their subsequent deeper sports participation behaviors.

Based on this, this paper proposes the research hypotheses:

*H3*: Perceived usefulness has a significant mediating effect between service personalization and sports participation.

*H4*: Perceived usefulness has a significant mediating effect between perceived value and sports participation.

### 3.4 The mediating role of exercise self-efficacy

In the interaction decision theory, self-efficacy holds that all human behaviors are governed by some kind of exchange activity that can bring rewards and remuneration (18). The application of smart sports technology is actually also an exchange, where the implementers of the technology aim to provide as much spiritual and material support as possible to customers in order to obtain rewards and social prestige. From this theoretical perspective, self-efficacy can influence human behavior, and behavior in turn affects self-efficacy (19), reflecting a positive relationship between the two.

In addition, self-efficacy has been proven in psychological research to have a negative predictive effect on symptoms such as depression and anxiety. After analyzing numerous domestic and international research results, Tang Zhengyu demonstrated that actively participating in regular exercise can effectively enhance an individual’s perception of physical activity ability, add a positive mood state, and increase satisfaction with life. Moreover, there is a mutual influence relationship between physical exercise and self-efficacy. Thus, it can be seen that self-efficacy and physical exercise are closely related to mental health, and they are the key factors (20) that promote the sustainability and longevity of self-exercise. Based on this, the research hypotheses are proposed as follows:

*H5*: Exercise self-efficacy has a significant mediating effect between service personalization and sports participation.

*H6:* Exercise self-efficacy has a significant mediating effect between perceived value and sports participation.

### 3.5 Chain mediating role of perceived usefulness and exercise self-efficacy

Based on the core framework of social cognitive theory, an individual’s efficacy expectation of the potential results of a behavior significantly shapes and strengthens their efficacy level. Specifically, in the context of smart sports applications, residents’ positive assessment of the perceived usefulness of technology constitutes a powerful efficacy expectation. This positive cognition of the utility of technology directly enhances their exercise self-efficacy by increasing their sense of control and the possibility of success in overcoming obstacles and completing exercise with the aid of this technological tool. As the core source of an individual’s internal motivation, exercise self-efficacy not only stimulates participation willingness but also enhances persistence in the face of difficulties, thereby directly driving more frequent, longer-lasting, and higher-quality sports participation behaviors.

Therefore, the high perceived value created by the service personalization feature of smart sports technology first acts on the user’s cognitive level, strengthening their positive judgment of the practicality of the technology; this strengthened efficacy expectation is then transformed into the user’s confidence in the combined efficacy of their own exercise ability and the technology; ultimately, this stable efficacy confidence is effectively converted into actual sports participation actions. This path reflects the progressive transmission mechanism from the cognitive level of technology value to the expectation of technology utility, and then to the individual’s belief in behavioral efficacy, ultimately leading to the target behavior. It reveals the internal psychological process by which smart sports technology influences residents’ exercise behaviors.

Based on this, the research hypothesis is proposed as follows:

*H7*: Perceived usefulness and exercise self-efficacy have a chain mediating effect between service personalization and perceived value and sports participation.

**Figure 1:**
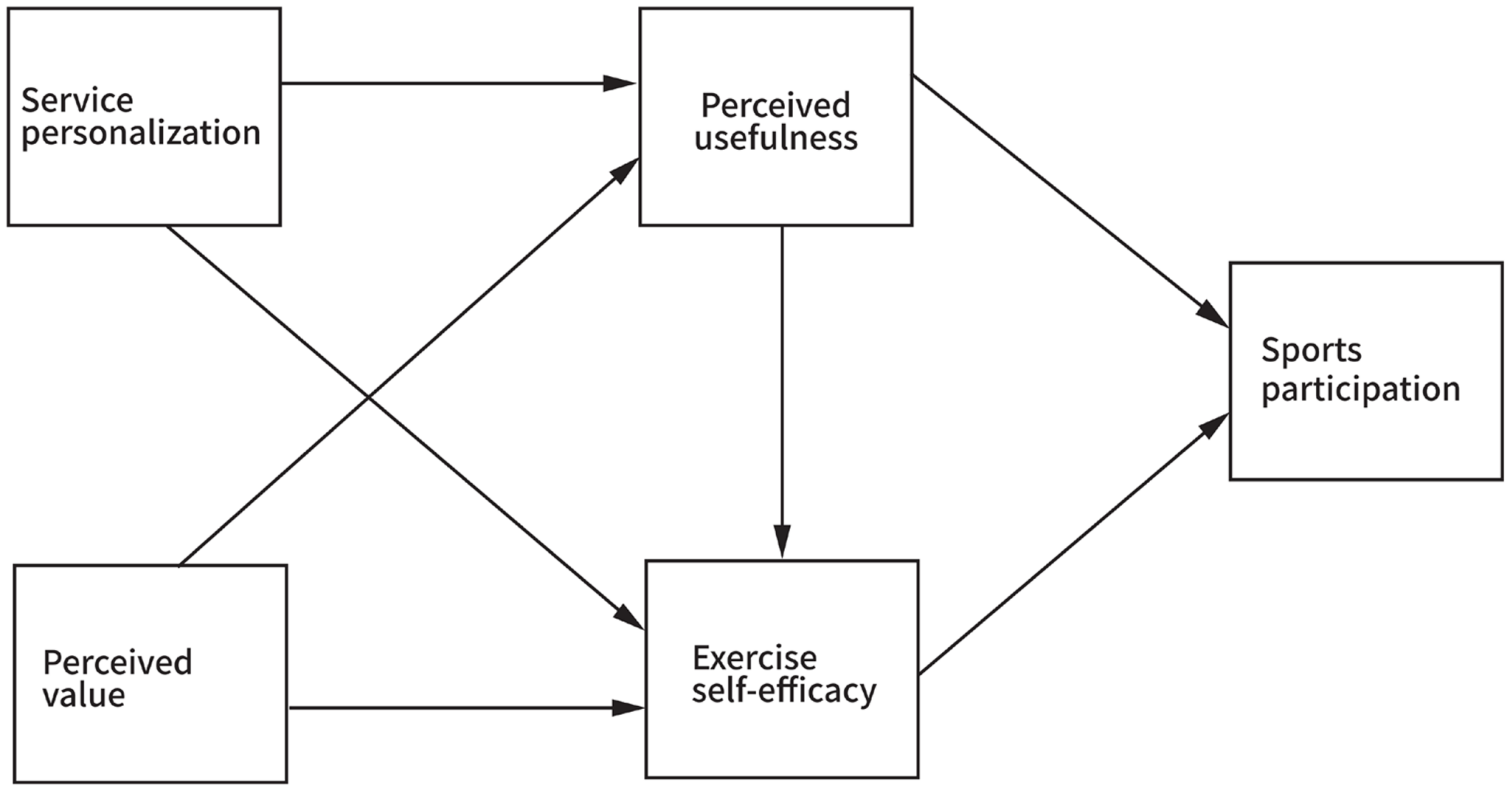
Hypothetical model of smart sports technology and sports participation.

In summary, this study will examine whether service personalization and perceived value in smart sports services have an impact on residents’ sports participation and explore the roles played by perceived usefulness and exercise self-efficacy in this process. The theoretical model is shown in Figure 1.

## 4 Research design

### 4.1 Data sources

This study collected research data through self-administered questionnaires, which were distributed online from June to August 2024. The distribution areas were selected from four first-tier cities with a high degree of application of smart sports technology: Beijing, Shanghai, Guangzhou, and Shenzhen. The respondents were required to be urban residents who had used smart sports technology services in the past year. To ensure the validity of the samples, the questionnaires were sent through the “Questionnaire Star” platform to the user groups of smart sports service partner communities and enterprises, and screening questions (such as “Have you used smart fitness equipment or related services?”) were set on the first page of the questionnaire. Only respondents who passed the screening could continue to fill it out, thereby ensuring the authenticity of the respondents’ identities. The final questionnaires were distributed in proportion to the population in each administrative district of the four cities, and a total of 354 valid questionnaires were collected, with an effective rate of 86.1%. The sample covered users of different ages, occupations, and technical adaptation levels from 18 to 65 years old, and was statistically representative.

### 4.2 Variable measurement

The core variables designed in this study include five variables: service personalization, perceived value, perceived usefulness, exercise self-efficacy, and sports participation. All scales in this study were based on mature scales developed by previous scholars and were appropriately modified according to the research needs. All items were measured using a five-point Likert scale, ranging from 1 (strongly disagree) to 5 (strongly agree).

Service personalization adopted a scale adapted from the scale development procedure proposed by Churchill (21) and modified by Zhou Tianshu (29). Since the main purpose of this study is to explore the impact of smart sports services on sports participation, from the perspective of consumers’ experience, a mature scale was selected and divided into 15 items for measurement, with a Cronbach’s Alpha value of 0.926.

In this study, to quantify the psychological factor indicator of perceived value, “social media” was selected as the carrier, and a mature scale was referred to, covering the three stages before, during, and after sports services. Therefore, perceived value was specifically refined into three dimensions: information creation and acquisition, interpersonal organization and interaction, and self and others’ emotions. The perceived value was measured using the questionnaire developed by Zhang Mingmin et al. (22), with a Cronbach’s Alpha value of 0.964.

Perceived usefulness referred to the questionnaire developed by Davis (23), with a Cronbach’s Alpha value of 0.902. Exercise self-efficacy was measured using the Exercise Self-Efficacy Scale (ESES) developed by Kroll et al. (24). The Cronbach’s Alpha value was 0.945.

The sports participation scale was adapted from the Deep Leisure Theory. Initially, Stebbins established six traits of deep leisure: perseverance, significant personal effort, lifelong pursuit, lasting benefits, unique character, and strong identification. Domestic scholars’ empirical research found that the results of this theory in the fields of tourism and culture could be well adapted to sports. Based on this, this study adopted the scale adapted by Chao Xu, identifying the six traits as perseverance, career, Unique temperament, Behavioral involvement, Long-term benefits, and strongly agree (25), with a total of 13 items and made adaptive adjustments to fit the research purpose. The Cronbach’s Alpha value was 0.959.

All scales were adapted through a rigorous process: (1) double-blind translation and back-translation to ensure semantic equivalence; (2) a pilot test with 50 smart sports service users, followed by interviews to assess item clarity and relevance. Based on pilot feedback, items were refined to enhance contextual fit.

It is worth noting that some scales exhibited Cronbach’s *α* values exceeding 0.95. In academic research, α values above 0.95 may suggest potential item redundancy. To address this concern, we re-examined the adapted scales and conducted a systematic scale refinement process. The procedure involved: (1) item-total correlation analysis to flag items with corrected item-total correlations greater than 0.85; (2) content overlap assessment by two independent researchers to identify semantic redundancy; (3) iterative exploratory factor analysis with stepwise item removal, retaining items that maintained factor loadings above 0.65 and did not increase cross-loadings; (4) comparative model testing to ensure that parsimony was not achieved at the expense of model fit.

Through this process, we removed 4 items from the Service Personalization scale, 4 items from the Perceived Value scale, 3 items from the Exercise Self-Efficacy scale, 2 items from the Perceived Usefulness scale, and 7 items from the Sports Participation scale. The final shortened scales demonstrated strong psychometric properties: all Cronbach’s *α* values ranged from 0.87 to 0.90; average variance extracted (AVE) values ranged from 0.54 to 0.59; composite reliability (CR) values ranged from 0.88 to 0.93; and the square roots of AVE exceeded all inter-construct correlations, confirming both convergent and discriminant validity, indicating that the scale refinement did not compromise construct validity.

### 4.3 Data analysis methods

The data analysis in this study was conducted in the following sequence: First, SPSS 27.0 and Amos 28.0 were used for reliability and validity analysis, and the common method variance was controlled using the latent variable error method. Second, the structural equation model combined with Bootstrap was used to test the chain mediation model. The structural equation model was constructed using Amos 28.0 to test the chain mediating effect of perceived usefulness and exercise self-efficacy between service personalization and sports participation. Finally, to enhance the robustness and accuracy of the research results, the Bootstrap method in PROCESS 4.12 was used for further verification of the research results.

## 5 Empirical results analysis

### 5.1 Structural equation model analysis

**Figure 2:**
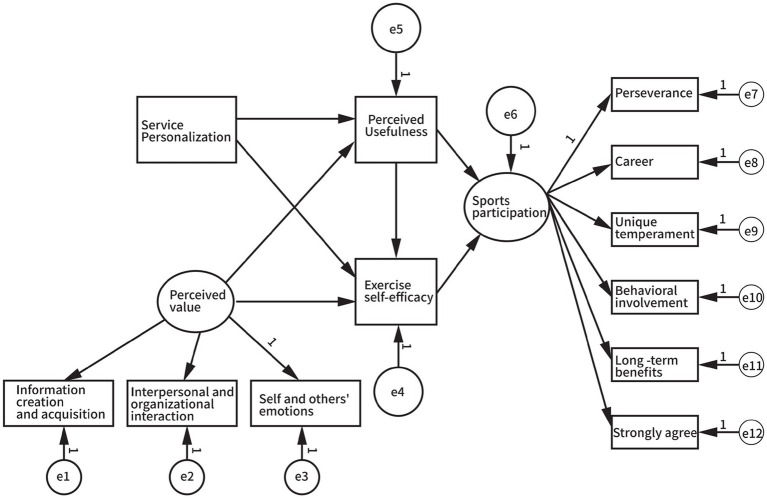
Structural equation modeling setup diagram.

Based on the structural equation hypothesis model shown in Figure 1 in the previous section, the perceived value in the model is mainly composed of three indicators: information creation and acquisition, interpersonal organizational and interaction, self and others’ emotions. Sports participation is mainly composed of six indicators: perseverance, career, unique temperament, behavioral involvement, long-term benefits, and strongly agree. Using Amos 28.0 software, the relationship model of the relevant variables was constructed through structural equation, as shown in Figure 2.

### 5.2 Descriptive statistical analysis

Table 1 presents the descriptive statistical results of the research sample in this study. Among them, there were 170 men (48.02%) and 184 women (51.98%). Regarding age, 44 people (12.43%) were under 20 years old, 109 people (30.79%) were between 21 and 30 years old, 103 people (29.1%) were between 31 and 40 years old, 48 people (13.56%) were between 41 and 50 years old, and 50 people (14.12%) were 50 years old or above. In terms of educational attainment, 49 people (13.84%) had an education level of junior high school or below, 20 people (5.65%) had a high school or technical secondary school education, 94 people (26.55%) had an associate degree, 172 people (48.59%) had a bachelor’s degree, and 19 people (5.37%) had a postgraduate degree or above. From the overall descriptive statistical distribution of the sample’s demographic characteristics, the proportion distribution of the sample data in this study is relatively reasonable and has a certain degree of data representativeness.

**Table 1:** Descriptive statistics table.

Name	Option	Number	Percentage
Sex	Male	170	48%
	Female	184	52%
Age	20 and below	44	12.4%
	21–30	109	30.8%
	31–40	103	29.1%
	41–50	48	13.6%
	50 and above	50	14.1%
Education	Junior high school and below	49	13.8%
	High school/Technical secondary school	20	5.6%
	Junior college	94	26.6%
	Bachelor’s degree	172	48.6%
	Postgraduate and above	19	5.4%
Total		354	100.0

Subsequently, the KMO sampling method and Bartlett’s sphericity test were employed to conduct factor analysis on the samples to determine their suitability. The specific analysis results are presented in Table 2.

**Table 2:** KMO result of questionnaire and Bartlett result.

Test method		Measured value
Kaiser–Meyer–Olkin sample measure		0.966
Bartlett’s test of sphericity	Approximate chi-square value	14615.252
	Degree of freedom	1,431
	Significance	0.000

From the test results, it can be found that the KMO value of the questionnaire in this study is 0.966 > 0.7, which indicates that it is suitable for factor analysis. The Bartlett’s sphericity test results show that the significance of the chi-square statistic is 0.000, which proves that the Bartlett’s sphericity test results are significant. Therefore, factor analysis can be further conducted.

### 5.3 Confirmatory factor analysis

#### 5.3.1 Reliability and validity tests

Table 3 presents the AVE and CR results of the model. Based on the calculation results of AVE and CR, it can reflect the combination reliability and convergent validity of the variables within the factor. Generally, if AVE is greater than 0.5 and CR is greater than 0.7, it indicates that the combination reliability and convergent validity are relatively good. In this study, the values of the average variance extracted (AVE) are all greater than 0.5, and the CR values are all greater than 0.7, which indicates that the extraction degree of the measurement indicators within the factor is excellent, and the convergent validity and combination reliability of the scale are both high.

**Table 3:** Combination reliability and aggregation validity test.

Measurement items	AVE	CR
Service personalization	0.610	0.926
Perceived value	0.608	0.959
Perceived usefulness	0.586	0.800
Exercise self-efficacy	0.612	0.940
Sports participation	0.621	0.955

#### 5.3.2 Discriminant validity test

Table 4 presents the results of the Pearson correlation analysis among factors and the square root of the average variance extracted (AVE). The square root of the AVE is used to indicate the strength of the internal correlation of a factor. If the square root of the AVE of a factor is greater than the Pearson correlation values with other factors, it indicates that the factor has relatively good discriminant validity. In this study, the correlation coefficients among several core variables are all less than the corresponding square root of the AVE, indicating that the core variables have a certain degree of correlation and a certain degree of distinction, and the discriminant validity of the scale is good.

**Table 4:** Correlation analysis and AVE mean root value.

Inter-item correlation matrix					
	_Service_ personalization	_Exercise_ self-efficacy	Perceived usefulness	Perceived value	Sports participation
_Service p_ersonalization	0.781	0.410	0.382	0.416	0.486
_Exercise_ self-efficacy	0.410	0.780	0.447	0.433	0.469
Perceived usefulness	0.382	0.447	0.766	0.436	0.488
Perceived value	0.416	0.433	0.436	0.782	0.439
Sports participation	0.486	0.469	0.488	0.439	0.788

### 5.4 Common method bias test

Using SPSS 27.0, this study conducted a common method bias test on the collected questionnaires. The Herman single-factor method was employed to examine the common method bias issue. The results revealed five factors with eigenvalues greater than 1, which together explained 65.939% of the total variance. The cumulative variance explained by the first factor was 20.070%. According to the current widespread application in the academic field, it is generally believed that the variance explained by a single factor should be less than 40% and not exceed half of the total variance explained. The results of this study conform to the above situation. Therefore, it can be concluded that this study has not been significantly affected by common method bias.

### 5.5 Model fit test

Table 5 presents the model fit indices, including common absolute fit indices: GFI, AGFI, and RM-SEA; incremental fit indices: NFI, RFI, IFI, TLI, and CFI; and parsimony fit indices: PCFI, PNFI, and *χ*^2^/df. The results show that GFI = 0.947; AGFI = 0.918; RM-SEA = 0.063; NFI = 0.970; RFI = 0.961; IFI = 0.983; TLI = 0.977; CFI = 0.983; PCFI = 0.744; PNFI = 0.735; *χ*^2^/df = 2.379.

**Table 5:** Model fitting index.

Fit indexes	Judgment criteria	Fit values	ULMC values	Differ
GFI	>0.90	0.947	0.953	0.006
AGFI	>0.90	0.918	0.925	0.007
RM-SEA	<0.08	0.063	0.049	0.014
NFI	>0.90	0.970	0.976	0.006
RFI	>0.90	0.961	0.969	0.008
IFI	>0.90	0.983	0.987	0.004
TLI	>0.90	0.977	0.981	0.004
CFI	>0.90	0.983	0.992	0.009
PCFI	>0.50	0.744	0.575	0.169
PNFI	>0.50	0.735	0.570	0.165
*x*^2^/df	<3.00	2.379	2.227	0.152

All the values in this study have reached their corresponding reference standards, indicating that the constructed model has a good fit with the sample data. At the same time, this also reflects that the model has a high overall explanatory power.

To further examine the potential influence of common method variance, we employed the Unmeasured Latent Method Construct (ULMC). A confirmatory factor analysis model was constructed including all trait factors (Service Personalization, Perceived Value, Perceived Usefulness, Exercise Self-efficacy, and Sports Participation), and an additional latent method factor was added. All items were allowed to load on both their respective trait factors and this method factor. Following recommended practices, the path coefficients from the method factor to each item were constrained to be equal to ensure model identification.

The results indicated that the model including the method factor showed a good fit (*χ*^2^/df = 2.227, CFI = 0.992, RMSEA = 0.049, GFI = 0.953, AGFI = 0.925). Compared to the measurement model without the method factor, the fit indices improved only slightly. As shown in Table 5, the changes in key fit indices were minimal:

ΔCFI = 0.009, ΔRMSEA = 0.014, ΔIFI = 0.004, and ΔTLI = 0.004 ΔTLI = 0.004, all of which are below the recommended thresholds. Although the parsimony fit indices (PCFI and PNFI) decreased due to the increased model complexity, the absolute fit indices remained excellent.

These results suggest that while a minimal amount of common method variance may be present, it does not pose a serious threat to the validity of our findings. The slight improvements in model fit after including the method factor corroborate the results of the Harman’s single-factor test, indicating that common method bias is not a major concern in this study.

### 5.6 Path analysis and hypothesis testing

**Figure 3:**
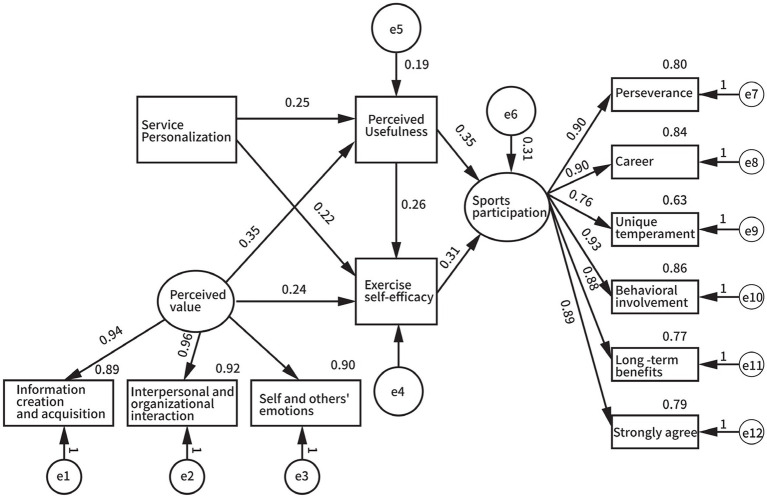
Model diagram of structural equations after standardized calculations.

Based on the structural equation model diagram set in Figure 2, the path relationship among the five factors in the model was tested using Amos 28.0. The relationships among the relevant variables were calculated, and the results are shown in Figure 3 and Table 6.

**Table 6:** Results of path relationship analysis of factors.

Path	Regression coefficient	Standardized regression coefficient	S. E.	C. R.	*p*
Service Personalization → Perceived usefulness	0.183	0.251	0.035	5.226	***
Service Personalization → Exercise self-efficacy	0.273	0.224	0.058	4.673	***
Perceived value → Exercise self-efficacy	0.479	0.241	0.101	4.764	***
Perceived value → Perceived usefulness	0.419	0.353	0.058	4.673	***
Perceived usefulness → Sports participation	0.121	0.348	0.018	6.874	***
Perceived usefulness → Exercise self-efficacy	0.479	0.264	0.101	4.764	***
Exercise self-efficacy → Sports participation	0.065	0.312	0.011	6.190	***
Sports participation → Perseverance	1.000	0.896			***
Sports participation → Career	1.440	0.919	0.052	27.752	***
Sports participation → Behavioral involvement	1.540	0.927	0.054	28.461	***
Sports participation → Long-term benefits	0.995	0.876	0.040	24.681	***
Sports participation → Unique temperament	0.501	0.793	0.025	20.036	***
Sports participation → Strongly agree	1.010	0.888	0.040	25.482	***
Perceived value → Self and others’ emotions	1.000	0.947			***
Perceived value → Interpersonal and organizational interaction	1.362	0.961	0.034	39.593	***
Perceived value → Information creation and acquisition	0.973	0.941	0.027	36.439	***

***The symbol indicates that the significance of this item is highly significant.

The results show that the direct effect of service personalization on sports participation (*β* = 0.491, 95% CI: 0.331–0.650) is significant and does not include 0, confirming the test results of the structural equation model, thus H1 is established. The direct effect of perceived value on sports participation (*β* = 0.168, 95% CI: 0.087–0.248) is significant and does not include 0, confirming the test results of the structural equation model, thus H2 is established.

The direct effect of service personalization on perceived usefulness (*β* = 0.251, *p* < 0.05), the direct effect of perceived usefulness on exercise self-efficacy (*β* = 0.264, *p* < 0.05), and the direct effect of exercise self-efficacy on sports participation (*β* = 0.312, *p* < 0.05) are all significant, thus the single mediating path effect of service personalization is significant, eliminating the interference of alternative paths. The direct effect of perceived value on perceived usefulness (*β* = 0.353, *p* < 0.05), the direct effect of perceived usefulness on exercise self-efficacy (*β* = 0.264, *p* < 0.05), and the direct effect of exercise self-efficacy on sports participation (*β* = 0.312, *p* < 0.05) are all significant, thus the single mediating path effect of perceived value is significant, eliminating the interference of alternative paths.

### 5.7 The test for mediating effect

Firstly, the Bootstrap mediating effect test method was adopted, with 5,000 repeated samplings, to further examine the chain mediating effect of perceived usefulness and exercise self-efficacy between smart sports technology and sports participation. When the 95% confidence interval does not include 0, it indicates a significant effect. The calculation results are shown in Table 7. Then, the mediating effect of perceived usefulness and exercise self-efficacy was tested through the structural equation model, and the Process program was used for further examination. SPSS 27.0 was used to test the chain mediating model of service personalization, perceived value, perceived usefulness, exercise self-efficacy, and sports participation. The calculation results are shown in Table 7.

**Table 7:** Results of bootstrap and process mediation tests of smart sports technology on sports participation.

Influencing path		Effect value	Standard	Bootstrap95%CI	
				Lower limit	Upper limit
Total effect	Service Personalization → Sports participation	0.831	0.079	0.674	0.987
	Perceived value → Sports participation	0.359	0.039	0.282	0.436
Direct effect	Service Personalization → Sports participation	0.491	0.081	0.331	0.650
	Perceived usefulness → Sports participation	0.168	0.041	0.087	0.248
Indirect effect	Service Personalization → Perceived usefulness	0.180	0.039	0.108	0.262
	Service Personalization → Exercise self-efficacy →Sports participation	0.109	0.028	0.057	0.169
	Service Personalization → Perceived usefulness → Exercise self-efficacy → Sports participation	0.050	0.015	0.024	0.085
	Perceived value → Perceived usefulness → Sports participation	0.102	0.021	0.062	0.144
	Perceived value → Exercise self-efficacy → Sports participation	0.060	0.015	0.033	0.092
	Perceived value → Perceived usefulness → Exercise self-efficacy → Sports participation	0.028	0.008	0.014	0.046

According to the results shown in Table 7, the indirect effect of service personalization → perceived usefulness → sports participation (*β* = 0.180, 95% CI: 0.108–0.262) and the indirect effect of service personalization → exercise self-efficacy → sports participation (*β* = 0.109, 95% CI: 0.057–0.169) are both significant. Since the direct effects in H1 and H2 are significant, it indicates that the mediating effect exists and is a partial mediating effect. The hypotheses H3 and H4, which state that perceived usefulness and exercise self-efficacy play a mediating role between service personalization and sports participation, are verified.

The indirect effect of perceived value → perceived usefulness → sports participation (*β* = 0.102, 95% CI: 0.062–0.144) and the indirect effect of perceived value → exercise self-efficacy → sports participation (*β* = 0.060, 95% CI: 0.033–0.092) are both significant. Since the direct effects in H1 and H2 are significant, it indicates that the mediating effect exists and is a partial mediating effect. The hypotheses H5 and H6, which state that perceived usefulness and exercise self-efficacy play a mediating role between perceived value and sports participation, are verified.

The total effect of service personalization → perceived usefulness → exercise self-efficacy → sports participation (*β* = 0.050, 95% CI: 0.024–0.085). In summary, the total indirect effect of service personalization is 0.339 (0.180 + 0.109 + 0.050), and the proportion of the chain path is approximately 14.7% (0.050/0.339).

The total effect of perceived value → perceived usefulness → exercise self-efficacy → sports participation (*β* = 0.028, 95% CI: 0.014–0.046). In summary, the total indirect effect of perceived value is 0.064 (0.021 + 0.015 + 0.028), and the proportion of the chain path is approximately 43.8% (0.028/0.064).

## 6 Discussion

### 6.1 Verification of the core path of the impact mechanism of smart sports technology

The research results show that, taking *χ*^2^/df, CFI, and RMSEA as examples, the fit values of 2.379, 0.983, and 0.063, respectively, indicate a high degree of fit between the structural equation model and the selected data, and the model test results are good. The path: service personalization → Perceived usefulness, *β* = 0.251, *p* < 0.001, indicates that the personality fit represented by service internalization is the fundamental driving force for forming the perception of the usefulness of technology (26). Combined with the significant paths of perceived usefulness → exercise self-efficacy (*β* = 0.264) and exercise self-efficacy → sports participation (*β* = 0.312), our results establish the complete sequential mechanism: personalization enhances usefulness perception, which strengthens efficacy belief, ultimately driving participation behavior. This chain mediation model provides empirical support for social cognitive theory’s application in the smart sports context.

Secondly, the path: Perceived usefulness → Sports participation, *β* = 0.348, *p* < 0.001, indicates that the perception of the usefulness of technology can directly stimulate participation behavior, echoing the social cognitive theory’s mention of the driving force of efficacy expectations, that is, the intensity of the motivation needed to stimulate and maintain behavior is influenced by the specific results and value expectations of that behavior. The path: Exercise self-efficacy → Sports participation, *β* = 0.312, *p* < 0.001, reflects that self-efficacy belief is the core psychological mechanism for maintaining long-term participation in sports exercise (27).

Beyond confirming previous findings, this study broadens the theoretical boundaries of prior research to some extent. First, by integrating TAM and SCT, we demonstrate that in the context of digital health interventions, the sequential mechanism of “perceived usefulness → exercise self-efficacy” provides stronger explanatory power for sustained behavioral engagement than either model alone. Specifically, the chain mediation model explains over 40% of the variance in sports participation. This suggests that in health-related technology contexts, cognitive evaluations (TAM) complemented by efficacy beliefs (SCT) will be helpful to fully capture the behavioral adoption process. Second, this study extends the boundary conditions of both theories by identifying “service personalization” as a critical environmental antecedent that activates the cognitive-affective pathway. While TAM traditionally focuses on perceived usefulness and ease of use as direct predictors, our findings reveal that these perceptions themselves are shaped by the degree to which technology adapts to individual needs. This refines our understanding of how technological features translate into user perceptions in personalized digital environments. Third, the study contributes to the digital health literature by demonstrating that the chain mediation mechanism operates differently across two distinct pathways: service personalization (technology-driven) and perceived value (experience-driven). The finding that service personalization has a stronger total effect than perceived value, suggests that in early-stage technology adoption, personalized features may be more critical than holistic value perceptions.

### 6.2 The interaction between service personalization and perceived value

In this study, the total effect values of service personalization and perceived value are 0.831 and 0.359 respectively, both showing significant positive influences. The effect value of service personalization is significantly higher than that of perceived value. Compared with perceived value (28), service personalization places more emphasis on the concept of “ownership” in the individual’s acceptance of sports services, that is, discovering how much of the service is truly “mine” in terms of cognition, and can also be regarded as a material guarantee for enhancing sports participation. This indicates that the degree of ownership of the service plays a crucial role in determining whether to continue participating in sports exercises, further confirming the conclusion from previous studies that perceived value does not directly affect service participation behavior. In other words, obtaining necessary service information is a prerequisite for forming a high level of perceived value, and a good perceived value is the basis for forming a sense of ownership of the service technology. Only when the two are organically combined and based on rational choice, will residents “buy into” smart sports services and personally participate in the exercises. Secondly, in the collected questionnaires, the average scores of the service personalization scale items “I can recognize what kind of service the enterprise or service personnel provide for me” and “During the service process, I feel the mastery and control of the service” are 3.36 and 3.38 respectively, which are higher than the average score of the items, providing data support for the statement that the higher the service control, the stronger the happiness obtained (29).

In addition, the path coefficient of interpersonal organization and interaction in perceived value is the largest, indicating that the preconditions influencing perceived value and the results of perceived value are closely related to personal emotional experience. That is to say, rich and positive emotional experiences can change an individual’s behavior patterns and enhance the connection between the individual and others in the process. Under this gradually formed influence, the individual’s willingness to continuously participate in sports exercises and loyalty are enhanced. Therefore, only when the residents participating in sports activities truly feel the satisfaction of interpersonal interaction and emotions during the enjoyment of sports services, can the digital technology service concept constructed by the sports platform truly take effect. This suggests that smart sports services should first be promoted in groups with the same goals and similar physical fitness levels, relying on the advantages of hierarchical structure, group regulations, and overall behavioral capabilities established within the group (30), and continuously publicized and radiated to informal groups, providing new options for creating a good reputation for benefits.

### 6.3 The chain transmission of exercise self-efficacy and perceived usefulness

The results of the mediation model test show that service personalization and perceived value can both positively associated with sports participation through the mediation of exercise self-efficacy and perceived usefulness, which further confirms the views of existing literature. The social ecological system theory (31) holds that an individual’s behavior is influenced by both cognitive factors and environmental perception factors. This study examines individual behavior within a multi-level system to explore the interaction between individual psychological variables and environmental variables. Through the mediation effect test, it is found that for every one-unit increase in the expected utility of smart sports technology, the belief in exercise efficacy increases by 0.312 units, and Service Personalization indirectly strengthens the belief in efficacy along this path (*β* = 0.224). This result demonstrates that service personalization and perceived value form a favorable cognitive assessment by promoting the improvement of technology functions, and ultimately internalize the exercise belief, thereby promoting sports participation.

Previous studies have also mentioned the influence of the external variable “social support” on physical exercise (32), that is, relatively stable material conditions, exercise environments, and emotional support can all contribute to creating a favorable external environment. This suggests that when applying perceived usefulness and ease of use, more attention should be paid to the more direct characteristics between perceived usefulness and usage intention, and multiple external environments should be considered to enhance perception. The more clear and concise the technical services provided are and the broader the content covered, the more users can recognize the important role and value of sports service technology. Additionally, it was found in the research that the impact of service personalization on perceived usefulness is slightly higher than its direct impact on exercise self-efficacy. This may imply that technical characteristics are more likely to influence cognitive assessment rather than directly change beliefs. This result reveals a subtle psychological mechanism, where Service Personalization directly acts on the cognitive level, and its obvious characteristics lead residents to have a preference in establishing usefulness, while also reflecting that the shaping of beliefs may require more mediating factors. The subtle differences shown in the above conclusions precisely confirm the above-mentioned transmission sequence of “environment → cognition → belief.” Overall, in the integration stage of smart sports services, it is necessary to make residents feel the advantages of the external environment in a more intuitive way, while also helping them shape a firm sports concept in a more delicate way.

### 6.4 Analysis of benefits of sports participation

As shown in Table 6, the coefficients of career and behavioral involvement are significantly higher than those of other dimensions, indicating that smart sports technology mainly promotes long-term sports investment and sports knowledge learning. This finding confirms the core view of the Health Action Process Approach theory, that is, when individuals enter the maintenance stage of behavior, continuous investment and knowledge internalization become the main characteristics (33). In contrast, the coefficient of unique temperament is the lowest, and its measurement items “mutual care and help” and “establishing common behavioral norms” reflect the construction of group identity, forming a theoretical connection with perceived value and perceived usefulness. This reveals an important rule: the cognitive intervention of smart sports mainly takes effect in the pre-participation decision-making stage, reducing the participation threshold through community influence; while after actual participation, users’ focus shifts to individual data feedback and practical value acquisition.

Based on this, this study innovatively discovers two mechanisms: First, unlike traditional health behavior models, the process of smart sports services presents a new path of “pre-participation group identity-driven → during participation individual data feedback → long-term self-efficacy reinforcement.” This indicates that relying on digital technology, through the improvement of resource allocation efficiency and the upgrading and reconstruction of sports products and services, the stage weights of health behaviors have been restructured (34). Secondly, residents’ behaviors such as adjusting training plans during injuries and persisting in exercise during low periods convert abstract beliefs into observable digital traces, providing an empirical basis for the quantitative research of sports exercise.

## 7 Conclusion

### 7.1 Research findings

This study, based on social cognitive theory and social ecological system theory, verified the multi-level mechanism through which smart sports technology influences sports participation. The research found that smart sports significantly promote users’ formation of technology utility cognition through the combination of service personalization and perceived value as environmental factors, thereby directly showing positive associations with sports participation behavior. This confirms the complete transmission path where technology characteristics influence behavior through cognitive assessment.

The structural equation model analysis indicates that the material guarantee function of service personalization is significantly superior to the emotional support function of perceived value, and interpersonal interaction constitutes the core dimension of perceived value. This establishes the key technical implementation path of group-level promotion. Participation benefits show distinct phased patterns: in the pre-participation stage, group identity construction is relied upon to lower participation barriers; in the during-participation stage, individual data feedback is used to maintain long-term commitment; in the post-participation stage, digital behavioral traces are utilized to strengthen beliefs, typically manifested as residents dynamically adjusting plans and maintaining exercise persistence in health challenges. In summary, a service innovation mechanism is proposed: moving the cognitive intervention window forward and quantifying behavior, thereby constructing a three-stage smart sports intervention framework based on group-driven, data-supported, and belief internalization.

### 7.2 Research limitations and prospects

(1) This study employed a cross-sectional design, which limits our ability to draw causal inferences. While structural equation modeling and mediation analysis reveal significant associations among variables that are consistent with theoretical predictions, these findings should be interpreted as evidence of association rather than causation. Mediation analysis in cross-sectional designs serves the essential first step of establishing whether variables relate to each other in theoretically expected patterns—a necessary precondition for subsequent causal testing. The significant path coefficients (service personalization → perceived usefulness *β* = 0.251; perceived usefulness → exercise self-efficacy *β* = 0.264; exercise self-efficacy → sports participation *β* = 0.312) and the significant chain mediation effect (*β* = 0.050) provide this foundational evidence by rejecting the null hypothesis of no association. However, we fully acknowledge that these findings do not establish temporal precedence or rule out reverse causality. To address this limitation, future research should adopt longitudinal designs (35) to examine the temporal relationships among variables, and experimental or quasi-experimental designs to establish causal effects. The theoretical model validated in this study provides clear hypotheses and testable pathways for such future causal investigations.

(2) In terms of sample representativeness, this study collected data exclusively from four first-tier cities in China (Beijing, Shanghai, Guangzhou, and Shenzhen). While these cities demonstrate a high degree of smart sports service application—serving as “pioneering frontiers” in the process of digital transformation—this geographical concentration may limit the generalizability of our findings to second- and third-tier cities or rural areas where smart sports infrastructure is less developed. It is important to clarify that the core objective of this study was to explore the core psychological mechanisms in the context of rapid urbanization and digital transformation. First-tier cities, with their advanced technological infrastructure and highly heterogeneous populations, provide an ideal “testing ground” to observe this mechanism with minimal interference from variables such as technology accessibility.

(3) In addition to the geographical concentration discussed above, the sample exhibits an imbalance in educational attainment that warrants further consideration. Specifically, 13.8% of respondents had an education level of junior high school or below, while only 5.6% had a high school or vocational education. This structural gap may reflect the characteristics of the questionnaire distribution in this study or the demographic profile of smart sports service users in first-tier cities.

Such educational imbalance may introduce systematic bias in the evaluation of key variables, particularly perceived usefulness. Compared to individuals with higher educational levels, users with lower educational attainment may interpret technological features differently, possess varying levels of digital literacy, or hold different expectations regarding technological utility. For instance, when judging whether a technology is useful, less educated users might rely more on intuitive experience rather than abstract functional assessments, potentially leading to differences in response patterns. Consequently, their evaluations of how useful smart sports services are for achieving exercise goals could be systematically shifted, which may in turn affect the observed relationships among variables. This bias may limit the generalizability of our findings to populations with different educational structures.

Future research should employ stratified sampling strategies to ensure a more balanced representation of educational levels. Additionally, qualitative studies could be conducted to explore how users with diverse educational backgrounds perceive and respond to the functional features of smart sports services, thereby providing deeper insights into the cognitive processes underlying technology adoption and verifying whether the chain mediation mechanism operates consistently across different educational strata.

## Data Availability

The original contributions presented in the study are included in the article/supplementary material, further inquiries can be directed to the corresponding author.
